# Supplementary material for: Reversing the charge transfer between platinum and sulfur-doped carbon support for electrocatalytic hydrogen evolution
Source: Nat Commun. 2019 Oct 31;10:4977. doi: 10.1038/s41467-019-12851-w (PMC6823491; doi:10.1038/s41467-019-12851-w)
Supplement: Supplementary file 1 — Supplementary Information [file 41467_2019_12851_MOESM1_ESM.pdf]

Supplementary Information

Reversing the charge transfer between platinum and sulfur-doped carbon support for electrocatalytic hydrogen evolution

Yan et al.

Supplementary Figures

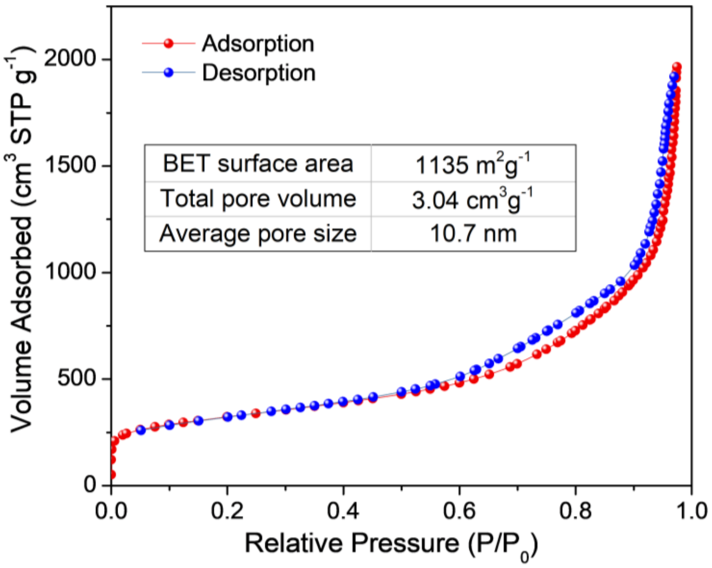

Supplementary Figure 1. N_2 adsorption-desorption isotherms of the S-C support.

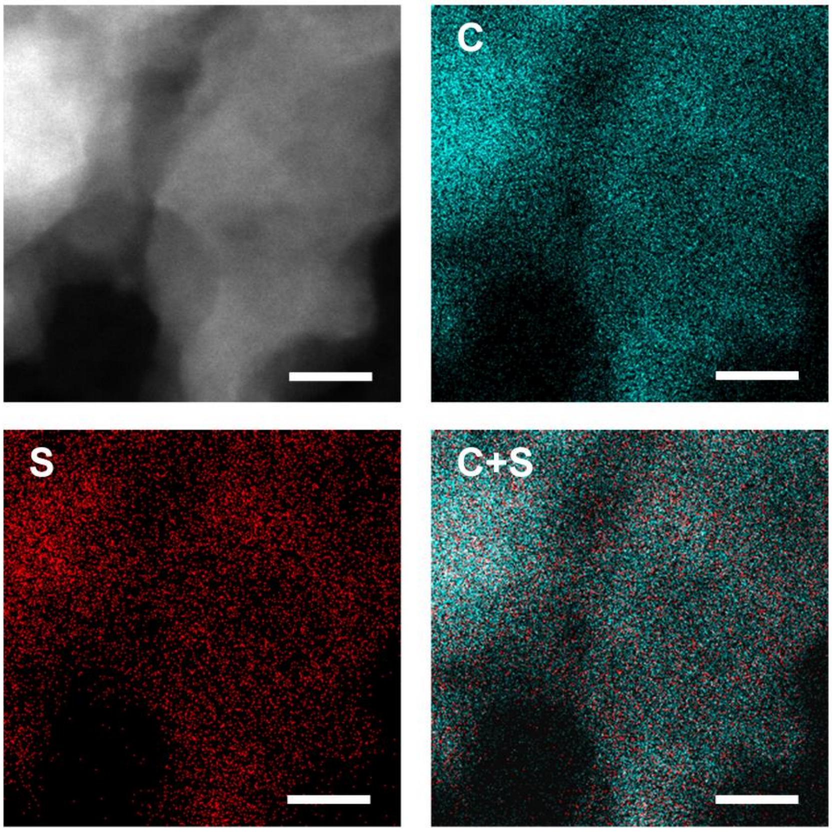

Supplementary Figure 2. HAADF-STEM image of S-C and corresponding elemental mapping. Scale bar, 10 nm. The images demonstrate that S is homogeneous distributed over the carbon support.

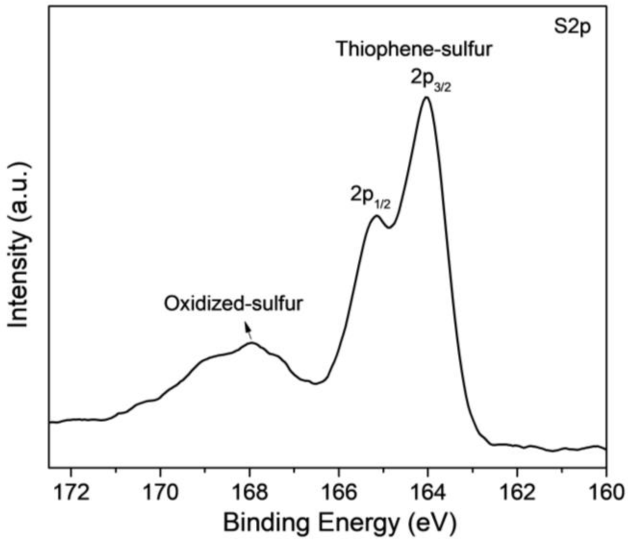

Supplementary Figure 3. High-resolution XPS-S2p spectra of the S-C support. The two peaks located at 164.0 and 165.1 eV can be assigned to C-S bonds, while the peak located at around 168.0 eV is belonging to S-O_x bonds.

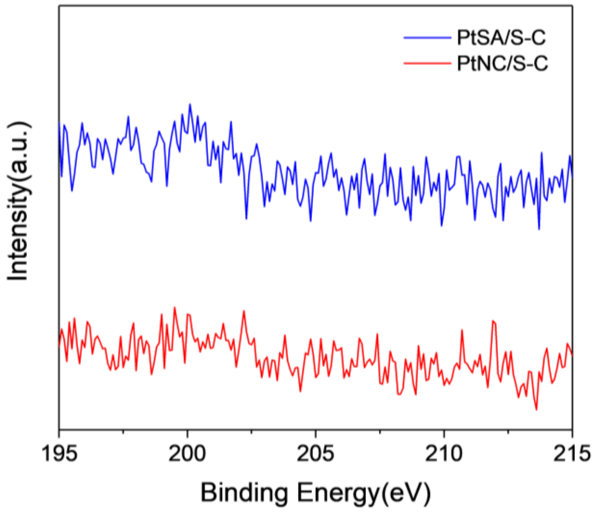

Supplementary Figure 4. Cl2p XPS spectra of PtSA/S-C and PtNC/S-C.

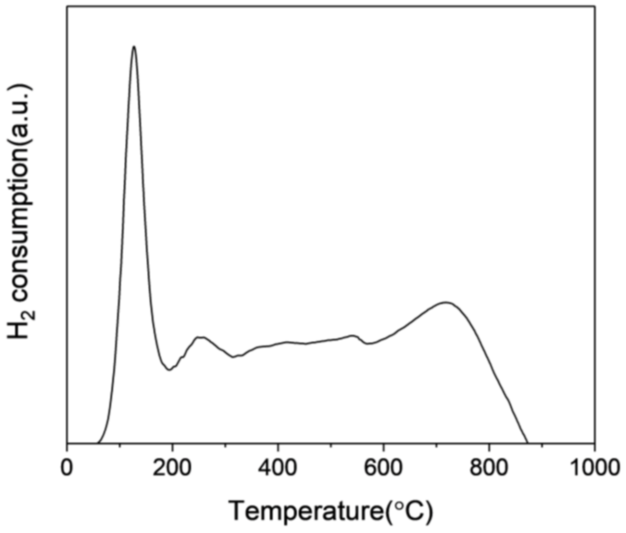

Supplementary Figure 5. Temperature-programmed reduction curve of H_2PtCl_6 on S-C.

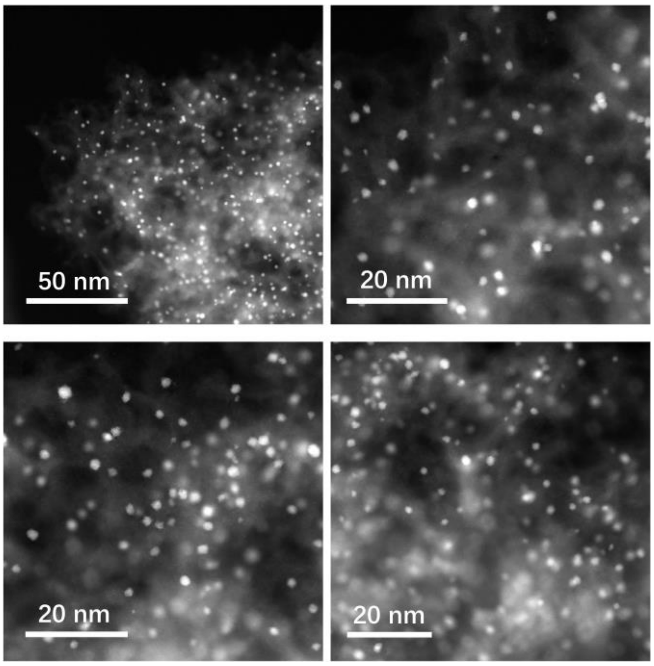

Supplementary Figure 6. Low-magnification HAADF-STEM images of PtNC/S-C.

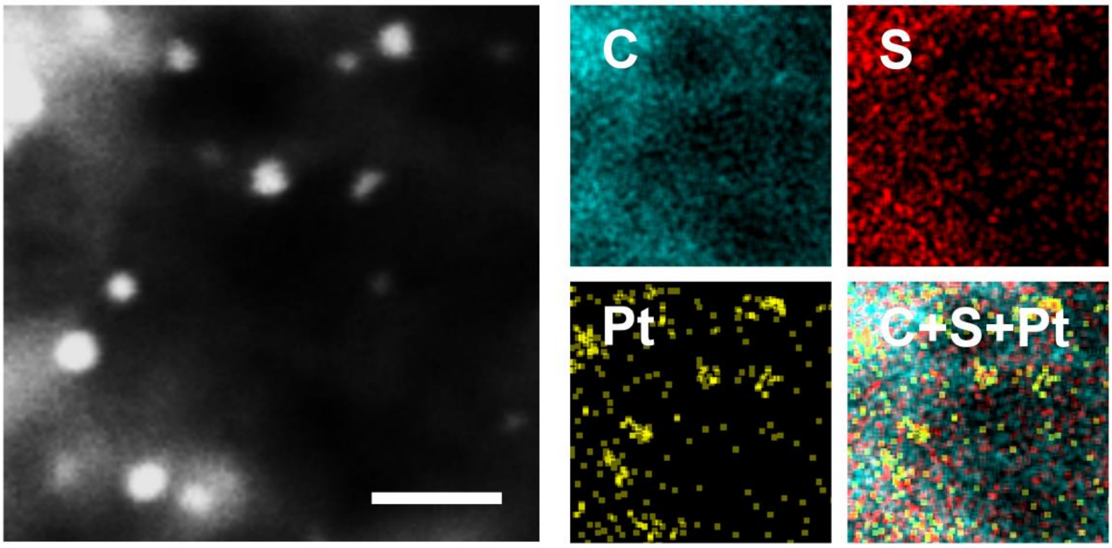

Supplementary Figure 7. HAADF-STEM image of PtNC/S-C and corresponding elemental mapping. Scale bar, 5 nm.

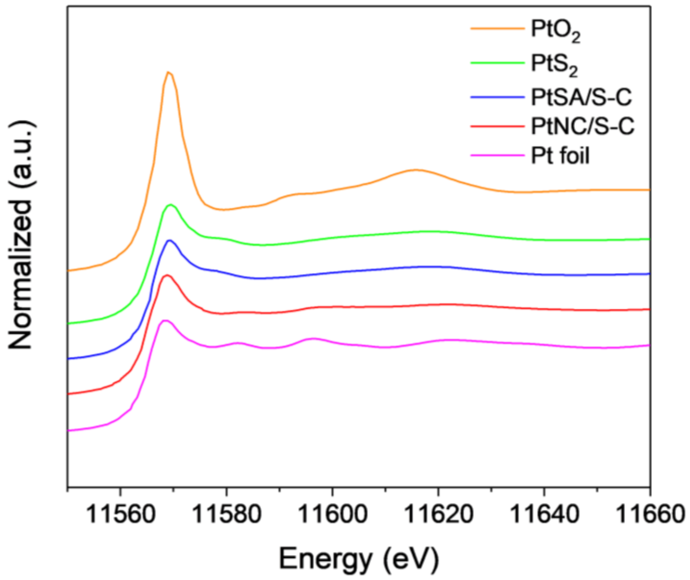

Supplementary Figure 8. Normalized XANES spectra at the Pt L_3-edge of PtSA/S-C, PtNC/S-C, PtO_2, PtS_2, and Pt foil.

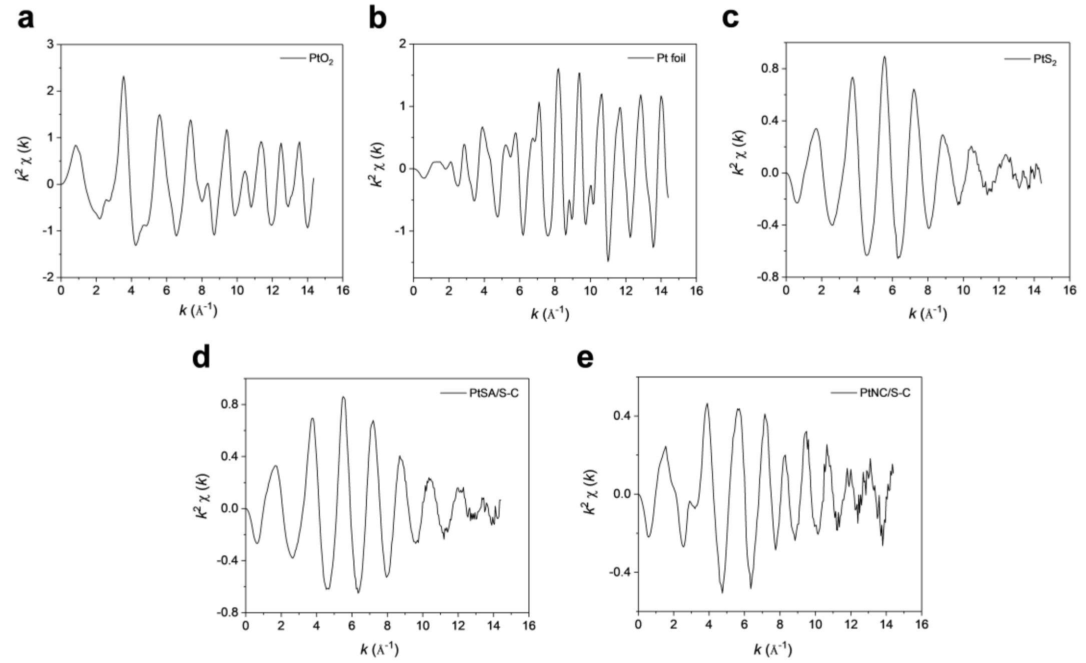

Supplementary Figure 9. *k* space of k^2-weighted Pt L_3-edge of (a) PtO_2, (b) Pt foil, (c) PtS_2, (d) PtSA/S-C and (e) PtNC/S-C.

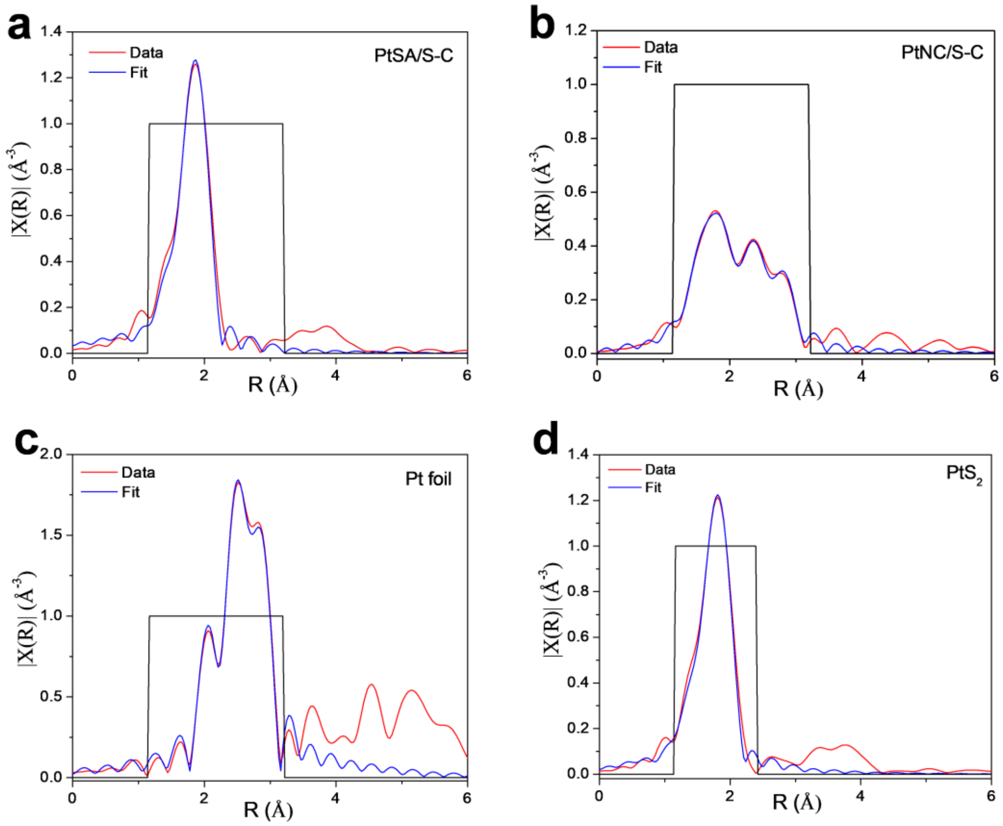

Supplementary Figure 10. The fit of Fourier transformed EXAFS spectra of Pt/S-C, Pt foil, and PtS_2. (a) PtSA/S-C. (b) PtNC/S-C. (c) Pt foil. (d) PtS_2.

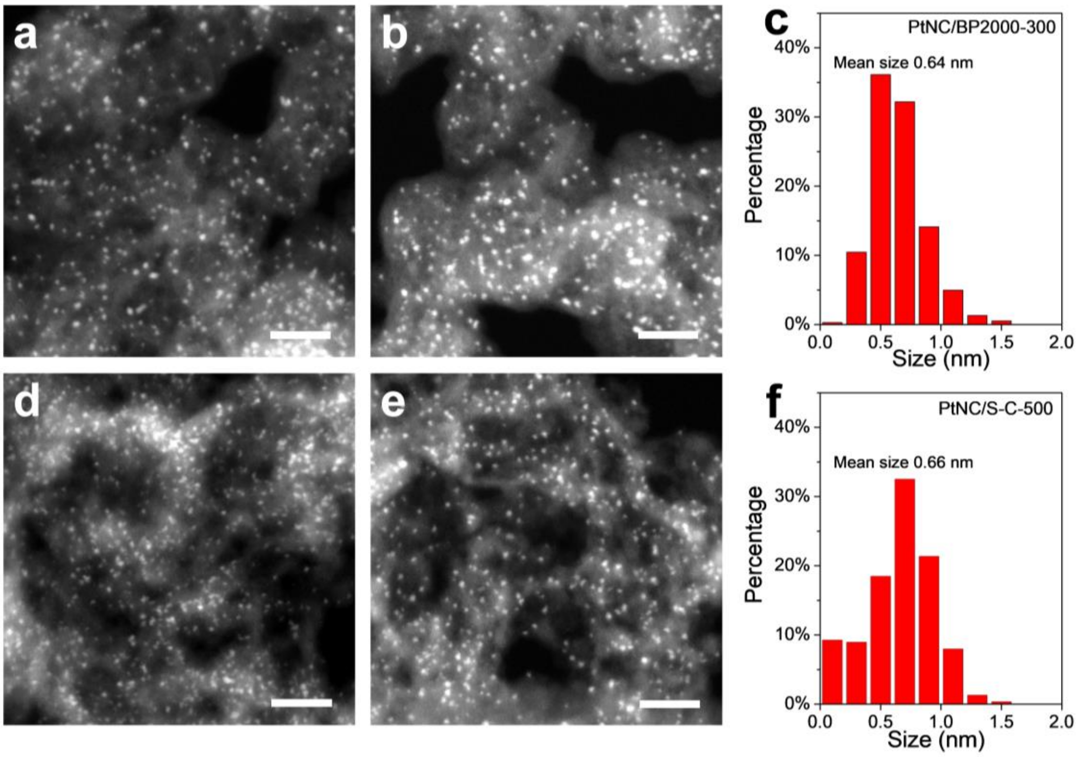

Supplementary Figure 11. HAADF-STEM characterization of PtNC/BP2000-300 and PtNC/S-C-500. (a,b) HAADF-STEM images of PtNC/BP2000-300 prepared at 300 °C. Scale bar, 10 nm. (c) Particle size distribution of PtNC/BP2000-300. d,e) HAADF-STEM images of PtNC/S-C-500 prepared at 500 °C. Scale bar, 10 nm. (f) Particle size distribution of PtNC/S-C-500. These results indicates the similar particle size of PtNC/BP2000-300 and PtNC/S-C-500.

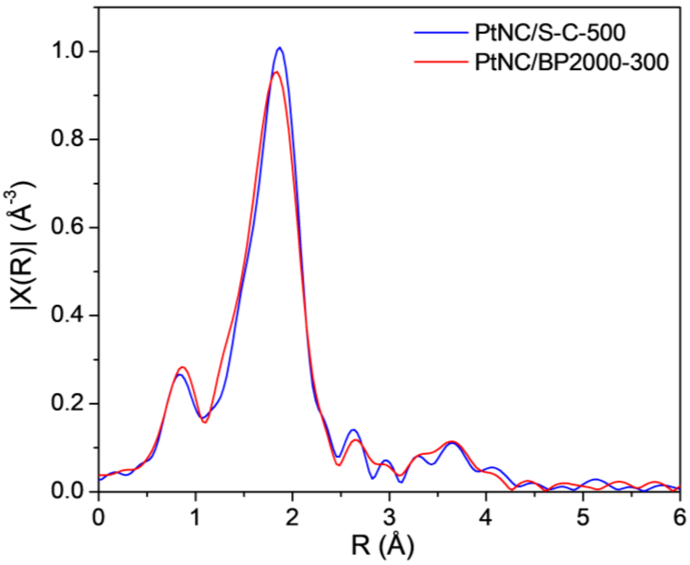

Supplementary Figure 12. Fourier transform of k^2-weighted Pt L_3-edge of PtNC/S-C-500 and PtNC/BP2000-300.

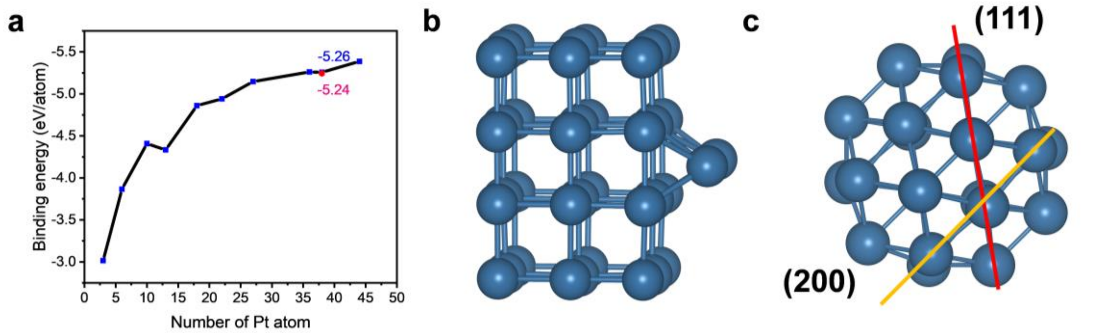

Supplementary Figure 13. Binding energy and structural configurations of Pt cluster. (a) Binding energy of a series of Pt_n cluster. The bind energy of cuboid-shape Pt_{38} and truncated octahedron Pt_{38} are marked with blue and red colors, respectively. The binding energy of Pt_n cluster is defined as E_b = E(system)/n, where n is the number of Pt atom. (b) Cuboid-shape Pt_{38} cluster. (c) Truncated octahedron Pt_{38} cluster with the zone axis [011] perpendicular to the paper. The red and yellow lines mark the crystal face (111) and (200) of Pt_{38} cluster, respectively.

The binding energy of a series of Pt clusters are showed in Supplementary Fig. 13a. The results indicate that the most stable configuration of Pt_{38} cluster is Pt_{36} cuboid capped with two Pt atoms on the 3x4 plane (Supplementary Fig. 13b). The binding energy of Pt_{38} with the shape of truncated octahedron (Supplementary Fig. 13c) is very close to that of cuboid-shape Pt_{38}, which means the former is also energy favored. These results are consistent with the Kumar's analyses[1]. Additionally, the HAADF-STEM image shows that the exposed crystal faces of PtNC/S-C prepared experimentally are (111) and (200) (Fig. 2c), which is in good agreement with the atomic array and exposed crystal plane of the proposed Pt_{38} structure. Overall, considering the computational and experimental results, we chose the Pt_{38} clusters with truncated octahedron shape in the current simulation work.

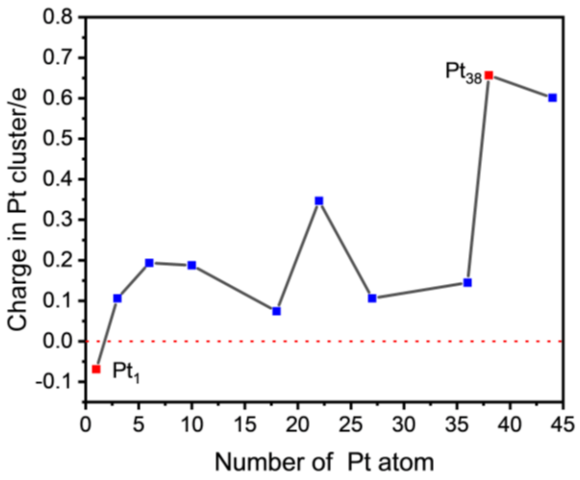

Supplementary Figure 14. Bader charge analysis results of a series of S-Graphene supported Pt_n cluster.

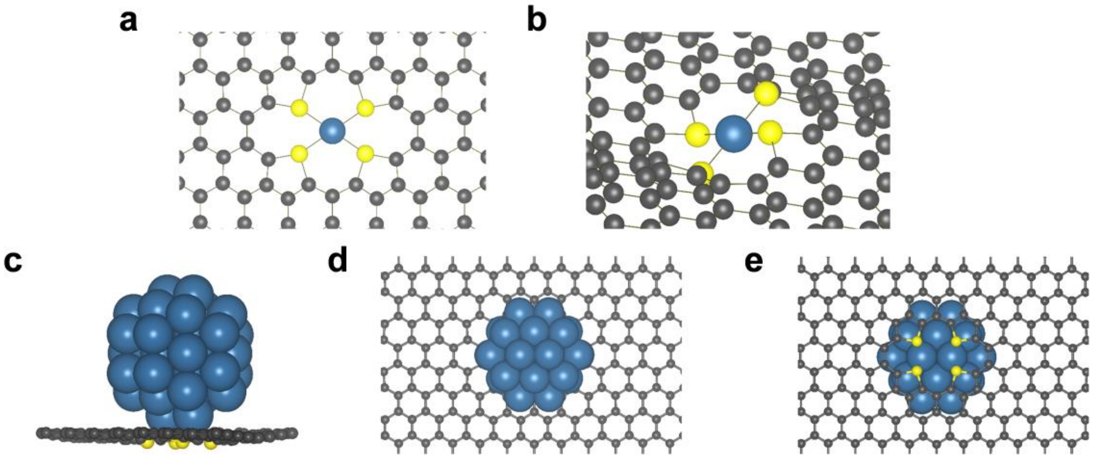

Supplementary Figure 15. Optimal model of Pt_1/S-Graphene and Pt_{38}/S-Graphene. (a) Top view of Pt_1/S-Graphene. (b) Side view of Pt_1/S-Graphene. (c) Side view of Pt_{38}/S-Graphene. (d) Top view of Pt_{38}/S-Graphene. (e) Bottom view of Pt_{38}/S-Graphene.

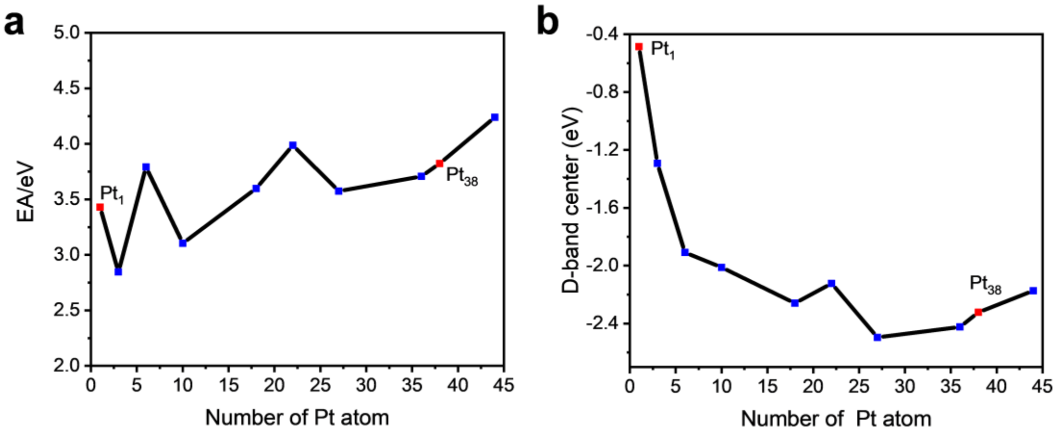

Supplementary Figure 16. (a) Electron affinity (EA, EA = E(Pt_n) – E(Pt_n^-)) and (b) d-band center of a series of Pt_n cluster.

We calculated the electron affinity of a series of Pt clusters from Pt_1 to Pt_{44} (Supplementary Fig. 16a). We found that the EA of Pt cluster increased slightly with the cluster size, which means that the ability of the Pt cluster system to capture electrons is enhanced. We further study the charge transfer by the density of state analyses for the Pt_1 and Pt_{38} (Supplementary Fig. 17). The d-orbital of Pt_1 is distributed between -1 and 0.5 eV, while the d-orbital of Pt_{38} clusters is clearly split with a wide distribution from -6 to +0.5 eV. The d-band center is used here to evaluate the degree of displacement of the d-orbit as the cluster size increases (Supplementary Fig. 16b). The d-band center of the Pt single atom is shallow, and its spin-dn d-orbital in PDOS (Supplementary Fig. 17a) is even located at the Fermi level. These d-orbital electrons should be easily to interact with the substrates. From Pt_1 to Pt_6, the d-band center decreases rapidly. The d-band center of large Pt_n cluster (n>6) is much more negative than that of Pt_1, indicating that the d-orbital of Pt_n cluster moves to a deeper level relative to Pt_1. The d-band center shift may be associated to the charge transfer from substrate to Pt cluster. Therefore, it can be concluded that the charge transfer between Pt and S-C is highly relevant to the platinum particle size, which is induced by the change of electron affinity as well as the shift of d-band center.

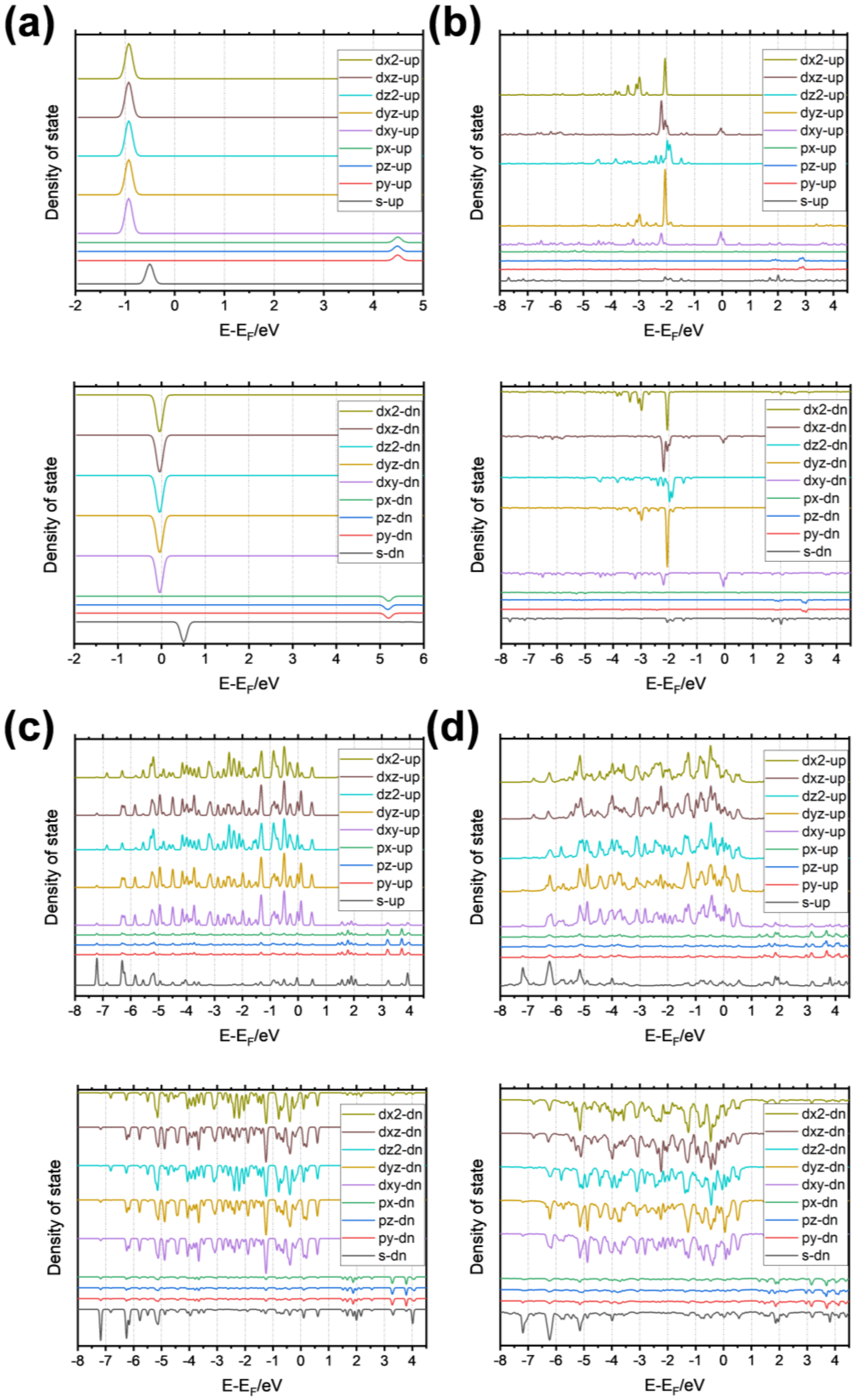

Supplementary Fig. 17. Projected density of states (PDOS) of (a) Pt_1, (b) Pt_1/S-Graphene, (c) Pt_{38} and (d) Pt_{38}/S-Graphene.

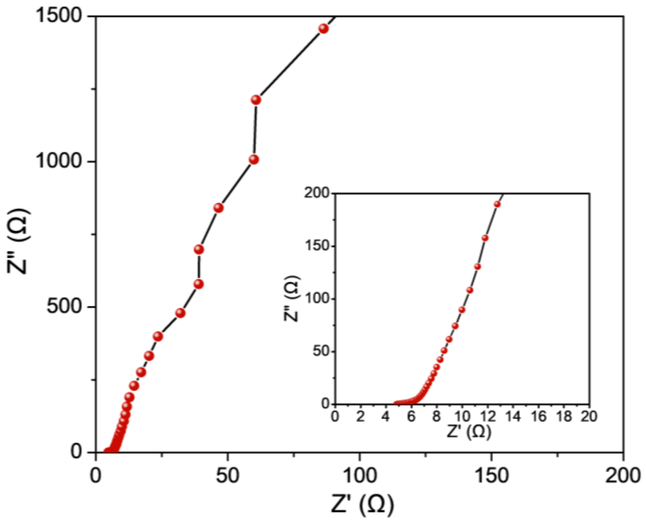

Supplementary Figure 18. Nyquist plots of PtNC/S-C in 0.5 M H_2SO_4. The solution resistances (Rs) are approximately 4.8 Ω in 0.5 M H_2SO_4.

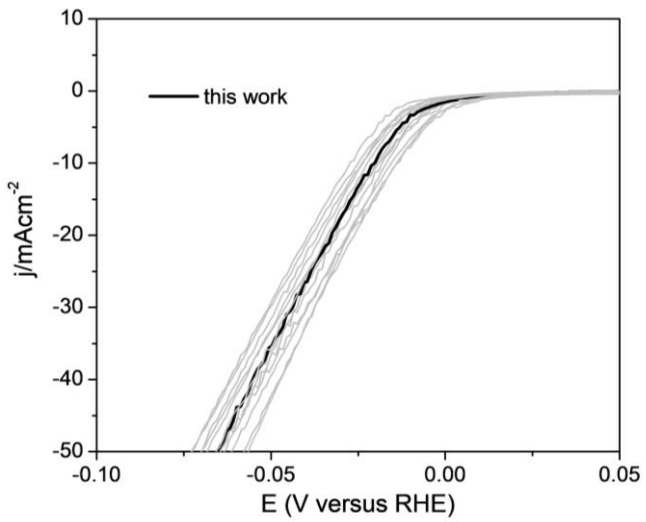

Supplementary Figure 19. The HER polarization curves of PtNC/S-C were carefully tested repeatedly for different batch samples. The polarization curve presented in this work was the middle activity of these curves (without IR correct).

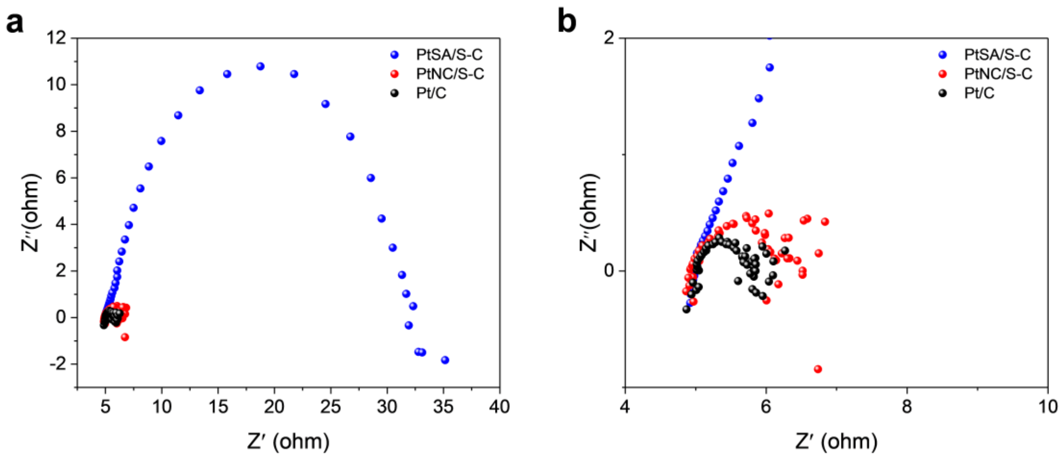

Supplementary Figure 20. Nyquist plots of Pt/S-C and commercial Pt/C.

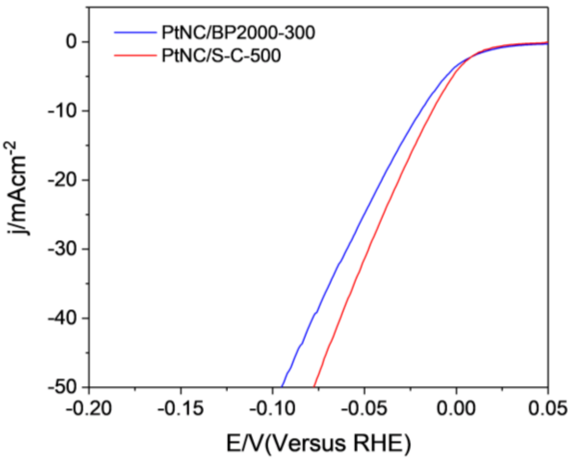

Supplementary Figure 21. HER activity of PtNC/BP2000-300 and PtNC/S-C-500 (without IR correct). In this measurement, the solution resistance was greatly reduced compared with the former samples, due to the use of luggin capillary.

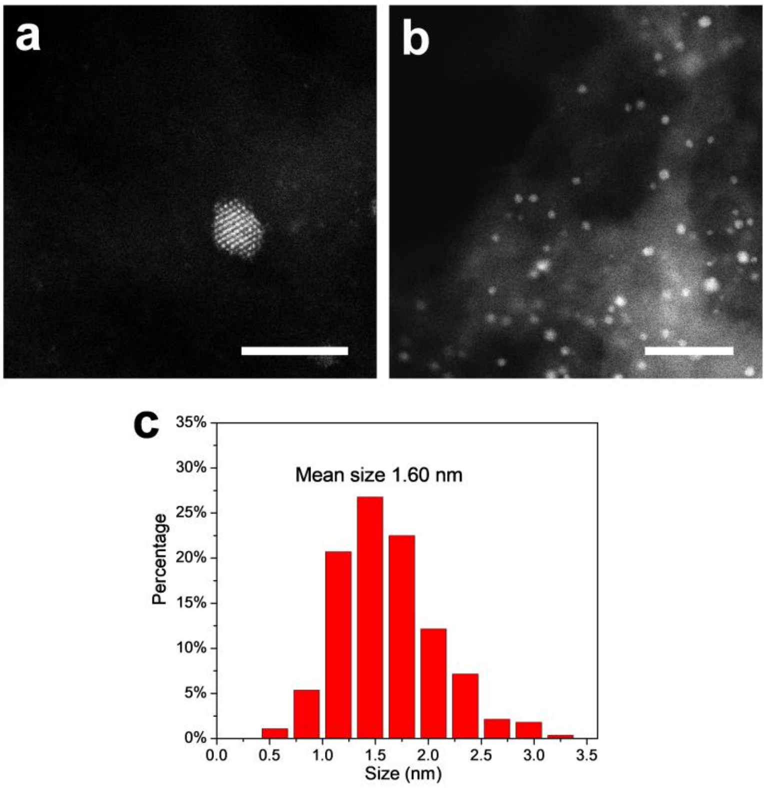

Supplementary Figure 22. (a,b) HAADF-STEM images of PtNC/S-C after ADT. Scale bar, 5 nm (a); 20 nm (b). (c) Histogram of particle size distribution of PtNC/S-C after ADT. The average particle size of PtNC/S-C after ADT is 1.60 nm, which is only slight larger than that of pristine PtNC/S-C.

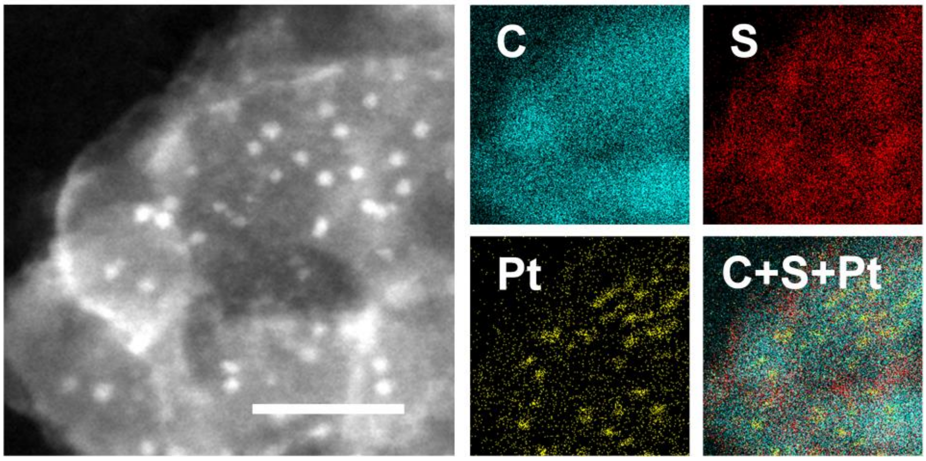

Supplementary Figure 23. HAADF-STEM image of PtNC/S-C after ADT and corresponding elemental mapping. Scale bar, 20 nm.

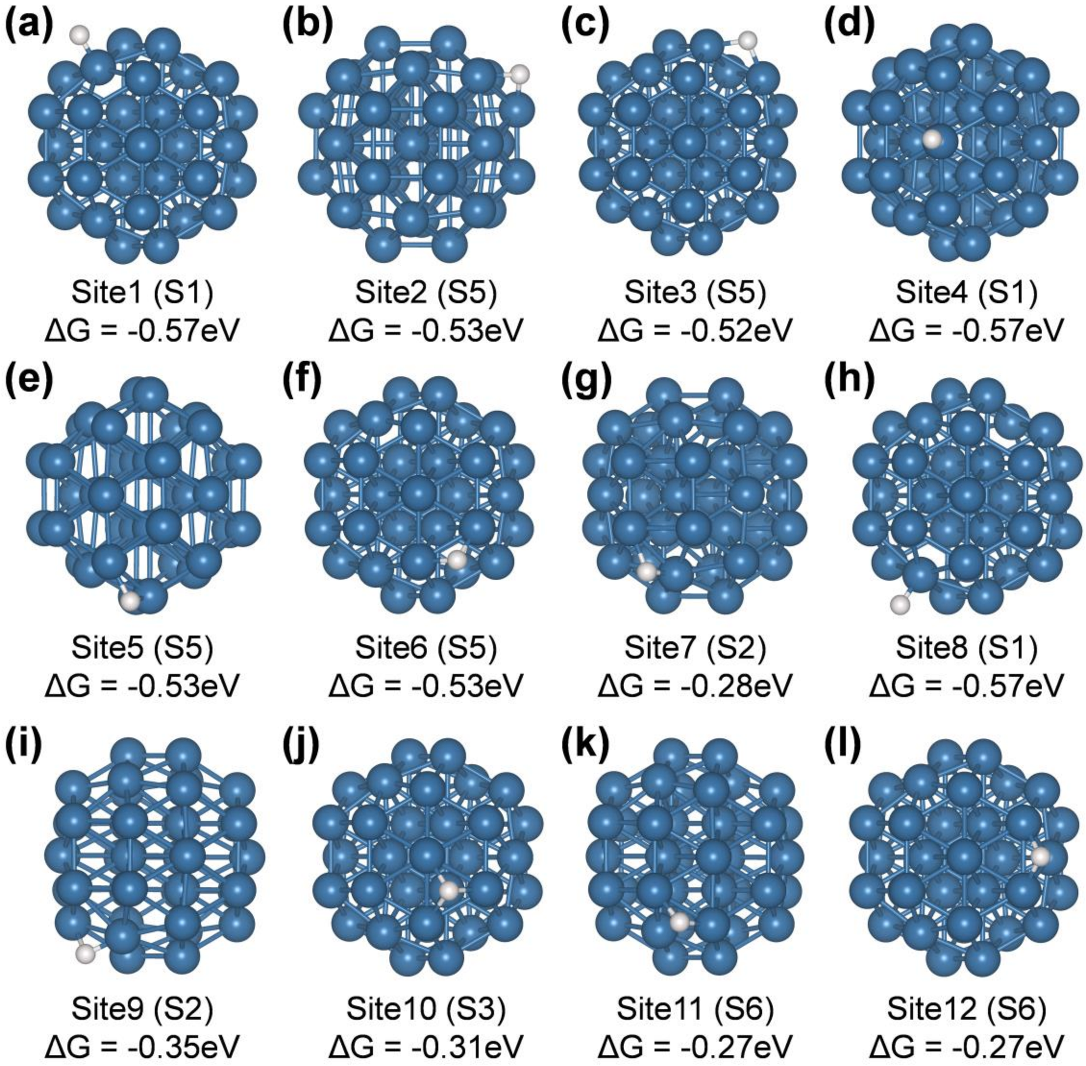

Supplementary Figure 24. Hydrogen absorption configuration and the corresponding free energy ΔG of free-standing Pt_{38}.

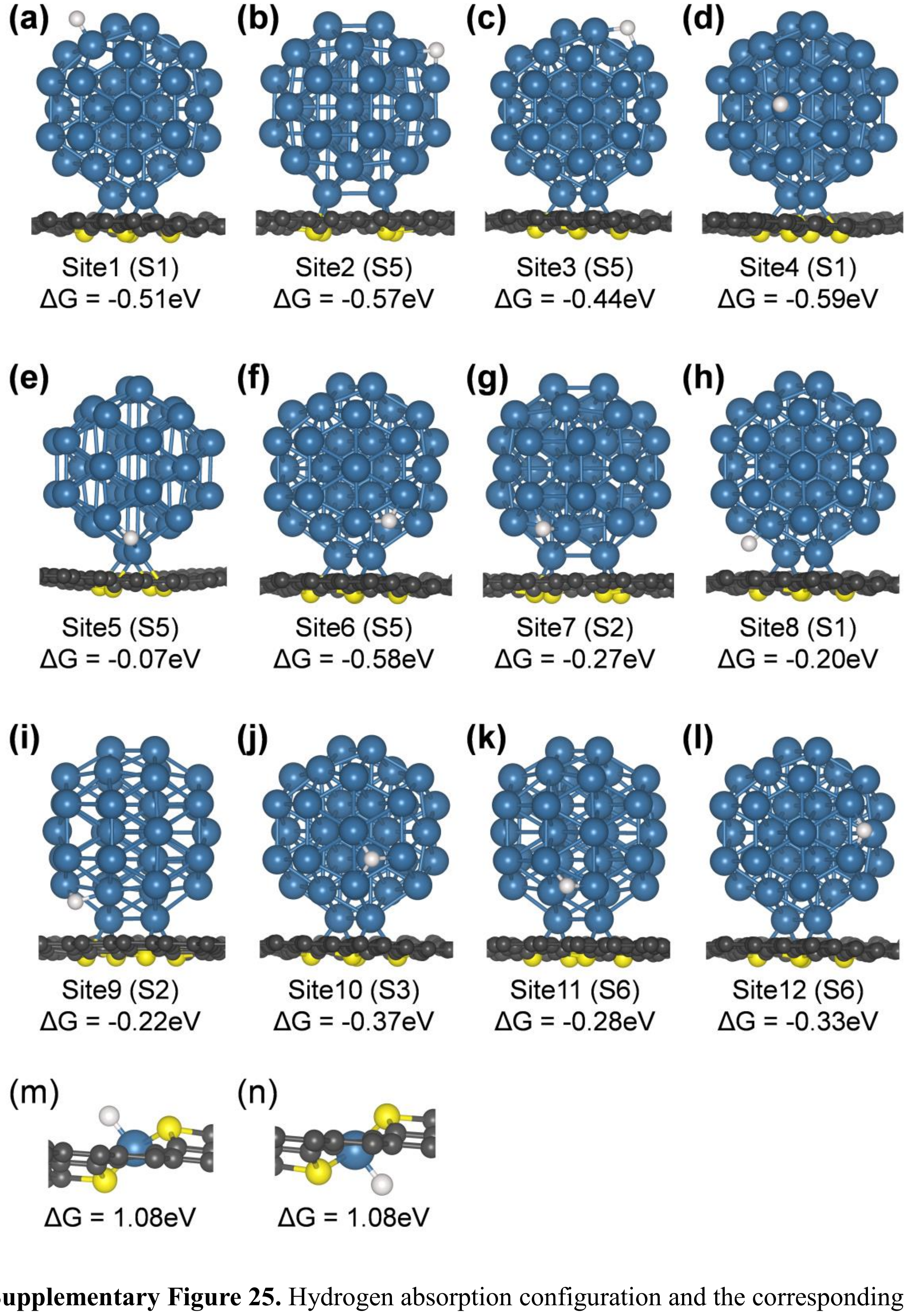

Supplementary Figure 25. Hydrogen absorption configuration and the corresponding free energy ΔG of Pt_{38}/S-Graphene (a-l) and Pt_1/S-Graphene (m,n).

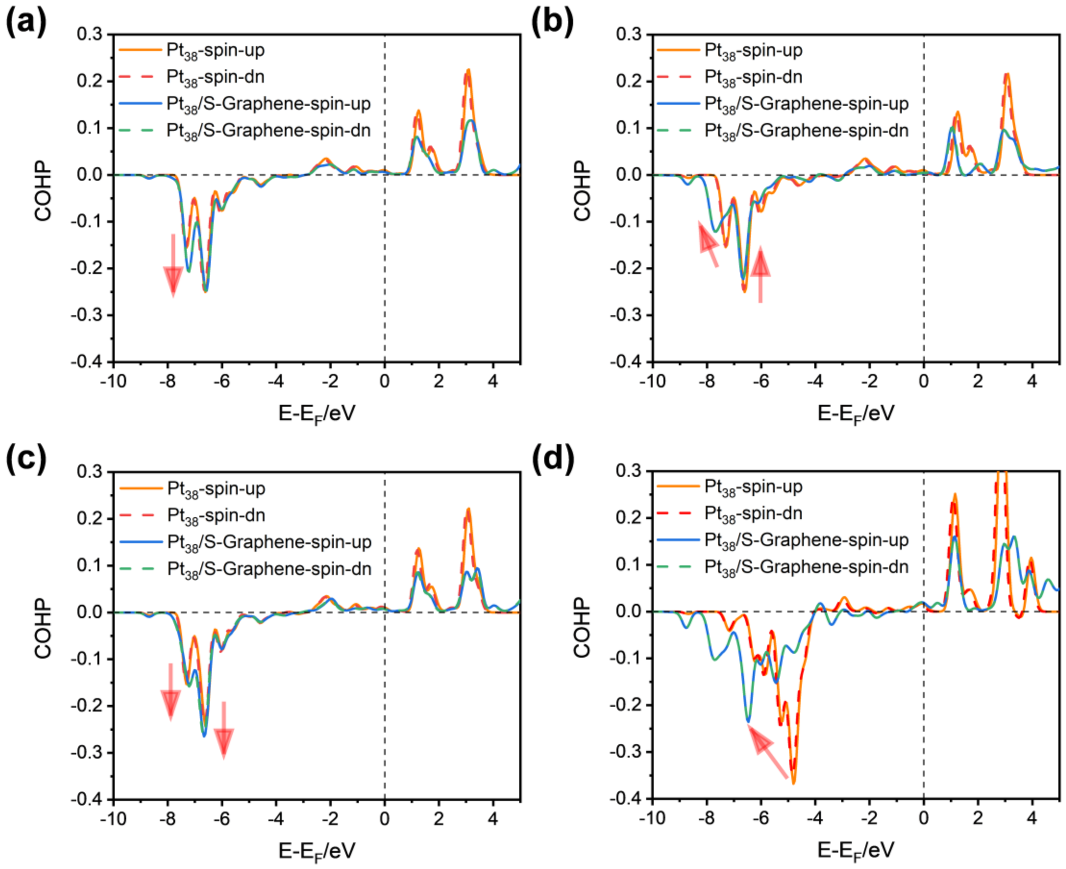

Supplementary Figure 26. Projected crystal orbital Hamilton populations (pCOHP) averaged over all Pt-H bonds: (a) site2, (b) site5, (c) site6, and (d) site8. pCOHP of spin-up and spin-dn are almost the same. The red arrow marks the change in peak intensity of Pt_{38}/S-Graphene relative to freestanding Pt_{38}.

We try to make further explanation about the δG by using COHP[2-4]. The negative and positive values of COHP represent the bonding interaction and anti-bond interaction between atoms, respectively. The COHP analysis results for two adsorption sites with negative δG (site2 and site6) and two adsorption sites with positive δG (site5 and site8) are shown in Supplementary Figure 26. The bonding interactions in the Pt-H bonds are mainly distributed in the range of -4 to -8eV. At site2 and site6, the peak intensity of bonding interaction increases, meaning that the bonding interaction in the Pt-H bond is enhanced, resulting in an increase adsorption strength of hydrogen at these sites. However, at the site5 and site8 sites, the peak intensity of bonding interaction decreases, which means that the bonding interaction in the Pt-H bond is weakened, resulting in weakening of the adsorption strength of hydrogen at these adsorption sites and leading to a positive δG value.

Supplementary Tables

Supplementary Table 1. Fitting results of R space of PtSA/S-C, PtNC/S-C, and reference samples.

Sample	Shell	N	R (Å)	σ^2 (10^{-3}Å^2)	ΔE_0 (eV)	R factor
PtSA/S-C	Pt-S	3.2±0.2	2.290±0.004	4.8±0.6	5.63±0.57	0.012
PtNC/S-C	Pt-S	1.8±0.4	2.260±0.016	10.1±3.4	1.42±1.50	0.005
	Pt-Pt	4.1±1.0	2.731±0.012	7.3±1.7	7.49±1.59	
PtS_2	Pt-S	3.5±0.2	2.258±0.005	6.3±0.6	2.22±0.60	0.007
Pt foil	Pt-Pt	12 (fixed)	2.764±0.002	4.5±0.3	8.48±0.39	0.003

Supplementary Table 2. Fitting results of R space of PtNC/S-C-500 and PtNC/BP2000-300, indicating that these two samples have close Pt-Pt coordination numbers and thus particle size.

Sample	Shell	N	R (Å)	σ^2 (10^{-3}Å^2)	ΔE_0 (eV)	R factor
PtNC/ S-C-500	Pt-S	3.291±0.388	2.274±0.148	7.2±1.2	4.75±1.57	0.017
	Pt-Pt	1.319±0.949	2.956±0.010	7.2±1.2	1.41±18.1	0.017
PtNC/ BP2000-300	Pt-C	4.566±0.672	2.037±0.031	0.26±1.5	7.62±2.11	0.007
	Pt-Pt	1.657±0.912	2.833±0.014	1.59±3.8	15.47±5.97	0.007

Supplementary Table 3. Comparison the HER performance of our catalyst with that of recently reported noble metal catalysts.

Catalyst	Catalyst loading amount (μg cm^{-2})	Overpotential at 10 mA cm^{-2} (mV)	Tafel Slope (mV $decade^{-1}$)	Ref.
**PtNC/S-C**	**2.55 (Pt)**	**11**	**24**	**This work**
Pt-GT-1	1.4 (Pt)	15	/	5
$Mo_2TiC_2T_x$–Pt_{SA}	12 (Pt)	30	30	6
Pt-Ni ASs	17 (Pt)	27.7	27	7
Pt SASs/AG	31.1(Pt)	12	29	8
Ru@C2N	81.8 (Ru)	13.5	30	9
Pt-MoS_2	27 (Pt)	53	40	10
Ru/GLC	40 (Ru)	35	46	11
Pt/NGNs	1.6 (Pt)	~38	29	12
Pt_3Ni_2 NWs-S/C	15.3 (Pt)	~28	/	13
Pt_3Ni_2 NWs/C-air	15.3 (Pt)	~34	/	14
Pt@DNA−GC	15 (Pt)	26	30	15
Pt-MoS_2	7.28 (Pt)	~38	25	16
RuP_2@NPC	233 (Ru)	38	38	17
NiAu/Au NPs	55.5 (Au)	~43	36	18
Pt_1/MC	10 (Pt)	~25	26	19
Pd/Cu-Pt nanorings	40.8 (Pd+Pt)	~22.8	25	20
400-SWNT/Pt	19.4 (Pt)	27	38	21
PtCoFe@CN	13.11 (Pt)	45	32	22
IrCo@NC-500	1.59 (Ir)	24	23	23
Ir@CON	102.5 (Ir)	13.6	27	24
Rh_2P	19.6 (Rh)	14	31.7	25
PtRu@RFCS-6h	15.6 (Pt+Ru)	19.7	27.2	26
Pt_1/NPC	3.8 (Pt)	25	28	27
Ru@GnP	80.25 (Ru)	13	30	28
L-RP	99.2 (Ru)	19	37	29
Pt_1/NMC	10 (Pt)	29	26	30

Supplementary Table 4. ΔG of Pt_{38}/S-Graphene, freestanding Pt_{38} and Pt_{38}-fix as well as δG, $δG_{geo}$ and $δG_{charge}$.

	**ΔG/eV**	**ΔG/eV**	**ΔG/eV**	**δG /eV**	**δGgeo/eV**	**δGcharge/eV**
**Absorption site**	**Pt_{38}/S-Graphene**	**Freestanding Pt_{38}**	**Pt_{38}-fix**	**Geometrical effect+charge**	**Geometrical effect**	**Charge**
Site1	-0.51	-0.57	-0.55	0.06	0.02	0.04
Site2	-0.57	-0.53	-0.56	-0.04	-0.03	-0.01
Site3	-0.44	-0.52	-0.49	0.08	0.03	0.05
Site4	-0.59	-0.57	-0.55	-0.02	0.02	-0.04
Site5	-0.07	-0.53	-0.45	0.46	0.08	0.38
Site6	-0.58	-0.53	-0.53	-0.05	0.00	-0.05
Site7	-0.27	-0.28	-0.32	0.01	-0.04	0.05
Site8	-0.20	-0.57	-0.39	0.37	0.19	0.18
Site9	-0.22	-0.35	-0.23	0.14	0.12	0.01
Site10	-0.37	-0.31	-0.33	-0.05	-0.02	-0.03
Site11	-0.28	-0.27	-0.22	0.00	0.05	-0.06
Site12	-0.33	-0.27	-0.29	-0.06	-0.02	-0.04

The δG is divided into two parts: $δG = δG_{geo} + δG_{charge}$, where $δG_{geo}$ and $δG_{charge}$ represent the influence of geometrical effect and charge transfer on the activity of Pt_{38}/S-Graphene, respectively. $δG_{geo}$ is defined as $δG_{geo}$ = ΔG(Pt_{38}-fix)-ΔG(Pt_{38}), where Pt_{38}-fix is Pt_{38} cluster by removing the substrate in Pt_{38}/S-Graphene and fixing all Pt atoms during structure relaxation. $δG_{charge}$ is defined as $δG_{charge}$ = ΔG(Pt_{38}/S-Graphene)-ΔG(Pt_{38}-fix). We tested 12 hydrogen adsorption sites (labeled as site1 to site12, respectively) and calculated the δG, $δG_{geo}$, and $δG_{charge}$ (Fig 6c and Supplementary Table 4). The $δG_{geo}$ of site8 and site9 are as large as 0.19 and 0.12 eV, respectively, which means that the geometric effect will also have an influence on the HER activity of Pt_{38}/S-Graphene. However, it should be noted that the charge transfer would affect the electronic structure of the system[31], and then induce the structural deformation. The structural deformation can in turn affect the charge transfer from the substrate to the Pt cluster by affecting its electronic structure. Although it is challenging to completely rule out the influence of geometric effect on the catalytic activity, it is safe to conclude that the outstanding HER performance of Pt-NC/S–C at some hydrogen absorption sites arise from the electron-enriched state of Pt, as a result of the size-dependent charge

transfer between Pt and the S–C support.

Reference

1. Kumar, V. & Kawazoe, Y. Evolution of atomic and electronic structure of Pt clusters: planar, layered, pyramidal, cage, cubic, and octahedral growth. *Phys. Rev. B* **77**, 205418 (2008).
2. Dronskowski, R. & Blöchl, P.E. Crystal orbital Hamilton populations (COHP): energy-resolved visualization of chemical bonding in solids based on density-functional calculations. *J. Phys. Chem.* **97**, 8617-8624 (1993).
3. Deringer, V.L., Tchougréeff, A.L. & Dronskowski, R. Crystal orbital Hamilton population (COHP) analysis as projected from plane-wave basis sets. *J. Phys. Chem. A* **115**, 5461-5466 (2011).
4. Maintz, S., Deringer, V.L., Tchougréeff, A.L. & Dronskowski, R. LOBSTER: A tool to extract chemical bonding from plane-wave based DFT. *J. Comput. Chem.* **37**, 1030-1035 (2016).
5. Tiwari, J.N.*, et al.* Multicomponent electrocatalyst with ultralow Pt loading and high hydrogen evolution activity. *Nat. Energy* **3**, 773-782 (2018).
6. Zhang, J.*, et al.* Single platinum atoms immobilized on an MXene as an efficient catalyst for the hydrogen evolution reaction. *Nat. Catal.* **1**, 985-992 (2018).
7. Zhang, Z.*, et al.* Crystal Phase and Architecture Engineering of Lotus-Thalamus-Shaped Pt-Ni Anisotropic Superstructures for Highly Efficient Electrochemical Hydrogen Evolution. *Adv. Mater.* **30**, 1801741 (2018).
8. Ye, S.*, et al.* Highly stable single Pt atomic sites anchored on aniline-stacked graphene for hydrogen evolution reaction. *Energy Environ. Sci.* **12**, 1000-1007 (2019).
9. Mahmood, J.*, et al.* An efficient and pH-universal ruthenium-based catalyst for the hydrogen evolution reaction. *Nat. Nanotech.* **12**, 441-446 (2017).
10. Huang, X.*, et al.* Solution-phase epitaxial growth of noble metal nanostructures on dispersible single-layer molybdenum disulfide nanosheets. *Nat. Commun.* **4**, 1444 (2013).
11. Chen, Z.*, et al.* Ruthenium/Graphene-like Layered Carbon Composite as an Efficient Hydrogen Evolution Reaction Electrocatalyst. *ACS Appl. Mater. Interfaces* **8**, 35132-35137 (2016).
12. Cheng, N.*, et al.* Platinum single-atom and cluster catalysis of the hydrogen evolution reaction. *Nat. Commun.* **7**, 13638 (2016).
13. Wang, P.*, et al.* Precise tuning in platinum-nickel/nickel sulfide interface nanowires for synergistic hydrogen evolution catalysis. *Nat. Commun.* **8**, 14580 (2017).
14. Wang, P., Jiang, K., Wang, G., Yao, J. & Huang, X. Phase and Interface Engineering of Platinum–Nickel Nanowires for Efficient Electrochemical Hydrogen Evolution. *Angew. Chem. Int. Ed.* **55**, 12859-12863 (2016).
15. Anantharaj, S., Karthik, P.E., Subramanian, B. & Kundu, S. Pt Nanoparticle Anchored Molecular Self-Assemblies of DNA: An Extremely Stable and Efficient HER Electrocatalyst with Ultralow Pt Content. *ACS Catal.* **6**, 4660-4672 (2016).

16. Chen, Z., *et al.* Interface confined hydrogen evolution reaction in zero valent metal nanoparticles-intercalated molybdenum disulfide. *Nat. Commun.* **8**, 14548 (2017).
17. Pu, Z., Amiinu, I.S., Kou, Z., Li, W. & Mu, S. RuP2-Based Catalysts with Platinum-like Activity and Higher Durability for the Hydrogen Evolution Reaction at All pH Values. *Angew. Chem. Int. Ed.* **56**, 11559-11564 (2017).
18. Lv, H., *et al.* A New Core/Shell NiAu/Au Nanoparticle Catalyst with Pt-like Activity for Hydrogen Evolution Reaction. *J. Am. Chem. Soc.* **137**, 5859-5862 (2015).
19. Wei, H., *et al.* Iced photochemical reduction to synthesize atomically dispersed metals by suppressing nanocrystal growth. *Nat. Commun.* **8**, 1490 (2017).
20. Chao, T., *et al.* Atomically Dispersed Copper–Platinum Dual Sites Alloyed with Palladium Nanorings Catalyze the Hydrogen Evolution Reaction. *Angew. Chem. Int. Ed.* **56**, 16047-16051 (2017).
21. Tavakkoli, M., *et al.* Electrochemical Activation of Single-Walled Carbon Nanotubes with Pseudo-Atomic-Scale Platinum for the Hydrogen Evolution Reaction. *ACS Catal.* **7**, 3121-3130 (2017).
22. Chen, J., *et al.* Enhanced Activity for Hydrogen Evolution Reaction over CoFe Catalysts by Alloying with Small Amount of Pt. *ACS Appl. Mater. Interfaces* **9**, 3596-3601 (2017).
23. Jiang, P., *et al.* Tuning the Activity of Carbon for Electrocatalytic Hydrogen Evolution via an Iridium-Cobalt Alloy Core Encapsulated in Nitrogen-Doped Carbon Cages. *Adv. Mater.* **30**, 1705324 (2018).
24. Mahmood, J., *et al.* Encapsulating Iridium Nanoparticles Inside a 3D Cage-Like Organic Network as an Efficient and Durable Catalyst for the Hydrogen Evolution Reaction. *Adv. Mater.* **30**, 1805606 (2018).
25. Yang, F., *et al.* A Monodisperse Rh2P-Based Electrocatalyst for Highly Efficient and pH-Universal Hydrogen Evolution Reaction. *Adv. Energy Mater.* **8**, 1703489 (2018).
26. Li, K., *et al.* Enhanced electrocatalytic performance for hydrogen evolution reaction through surface enrichment of platinum nanocluster alloying with ruthenium in-situ embedded in carbon. *Energy Environ. Sci.* **11**, 1232-1239 (2018).
27. Li, T., Liu, J., Song, Y. & Wang, F. Photochemical Solid-Phase Synthesis of Platinum Single Atoms on Nitrogen-Doped Carbon with High Loading as Bifunctional Catalysts for Hydrogen Evolution and Oxygen Reduction Reactions. *ACS Catal.*, 8450-8458 (2018).
28. Li, F., *et al.* Mechanochemically Assisted Synthesis of a Ru Catalyst for Hydrogen Evolution with Performance Superior to Pt in Both Acidic and Alkaline Media. *Adv. Mater.* **30**, 1803676 (2018).
29. Yu, J., *et al.* Bigger is Surprisingly Better: Agglomerates of Larger RuP Nanoparticles Outperform Benchmark Pt Nanocatalysts for the Hydrogen Evolution Reaction. *Adv. Mater.* **30**, 1800047 (2018).
30. Wei, H., *et al.* Ultralow-temperature photochemical synthesis of atomically

dispersed Pt catalysts for hydrogen evolution reaction. *Chem. Sci.* **10**, 2830-2836 (2019).
31. Meng, Y.-S., Sato, O. & Liu, T. Manipulating Metal-to-Metal Charge Transfer for Materials with Switchable Functionality. *Angew. Chem. Int. Ed.* **57**, 12216-12226 (2018).
